# Desigualdades en la salud maternoinfantil de los migrantes: el caso de Haití y la República Dominicana^*^

**DOI:** 10.26633/RPSP.2021.100

**Published:** 2021-09-16

**Authors:** Roberta Bouilly, Giovanna Gatica-Domínguez, Marilia Mesenburg, Francisco I. Cáceres Ureña, Daniel G. P. Leventhal, Aluísio J. D. Barros, Cesar G. Victora, Fernando C. Wehrmeister

**Affiliations:** 1 Universidad Federal de Pelotas Pelotas Brasil Universidad Federal de Pelotas, Pelotas, Brasil.; 2 Universidad Federal de Ciencias de la Salud Porto Alegre Brasil Universidad Federal de Ciencias de la Salud, Porto Alegre, Brasil.; 3 Universidad Autónoma de Santo Domingo Santo Domingo República Dominicana Universidad Autónoma de Santo Domingo, Santo Domingo, República Dominicana.; 4 Oficina Nacional de Estadística Santo Domingo República Dominicana Oficina Nacional de Estadística, Santo Domingo, República Dominicana.

**Keywords:** Migración humana, salud materna, salud del niño, disparidades en atención de salud, Haití, República Dominicana, Human migration, maternal health, child health, healthcare disparities, Haiti, Dominican Republic, Migração humana, saúde materna, saúde da criança, disparidades em assistência à saúde, Haiti, República Dominicana

## Abstract

**Objetivo.:**

Evaluar la cobertura y las desigualdades en las intervenciones de salud maternoinfantil entre haitianos, migrantes haitianos en la República Dominicana y dominicanos.

**Métodos.:**

Estudio transversal con datos de encuestas representativas en el nivel nacional realizadas en Haití en el 2012 y en la República Dominicana en el 2014. Se compararon nueve indicadores: la demanda de planificación familiar satisfecha con métodos modernos, la atención prenatal, la atención del parto (por personal de salud calificado), la vacunación infantil (con vacuna con la tuberculosis, el sarampión y tres dosis de la vacuna triple bacteriana), la gestión de casos de enfermedad en la infancia (administración de sales de rehidratación oral para la diarrea y búsqueda de atención sanitaria ante la sospecha de neumonía) e índice de cobertura compuesto. La riqueza se midió mediante un índice basado en los activos, dividido en terciles, y el lugar de residencia (urbano o rural) se determinó según la definición del país.

**Resultados.:**

La población haitiana mostró la menor cobertura respecto de la demanda de planificación familiar satisfecha con métodos modernos (44,2%), atención prenatal (65,3%), asistencia calificada en el parto (39,5%) y búsqueda de atención sanitaria ante la sospecha de neumonía (37,9%), y la mayor cobertura respecto de la administración de sales de rehidratación oral para la diarrea (52,9%); los migrantes haitianos presentaron la menor cobertura en DPT3 (44,1%) y la administración de sales de rehidratación oral para la diarrea (38%) y la mayor cobertura en la búsqueda de atención sanitaria ante la sospecha de neumonía (80,7%). La población dominicana presentó la cobertura más alta en la mayoría de los indicadores, excepto en la administración de sales de rehidratación oral para la diarrea y en la búsqueda de atención sanitaria ante la sospecha de neumonía. El índice de cobertura compuesto fue de 79,2% para los dominicanos, 69,0% para los migrantes haitianos y 52,6% para los haitianos. Las desigualdades socioeconómicas tuvieron, en general, un patrón a favor de los ricos y de las zonas urbanas en todos los grupos analizados.

**Conclusión.:**

Los migrantes haitianos en la República Dominicana presentaron una mayor cobertura que la población haitiana residente en Haití, pero menor que la población dominicana. Ambos países deberían planificar acciones y políticas para aumentar la cobertura y abordar las desigualdades existentes en las intervenciones de salud materna.

## Abstract (en)

**Objective.:**

To assess coverage and inequalities in maternal and child health interventions among Haitians, Haitian migrants in the Dominican Republic and Dominicans.

**Methods.:**

Cross-sectional study using data from nationally representative surveys carried out in Haiti in 2012 and in the Dominican Republic in 2014. Nine indicators were compared: demand for family planning satisfied with modern methods, antenatal care, delivery care (skilled birth attendance), child vaccination (BCG, measles and DPT3), child case management (oral rehydration salts for diarrhea and careseeking for suspected pneumonia), and the composite coverage index. Wealth was measured through an asset-based index, divided into tertiles, and place of residence (urban or rural) was established according to the country definition.

**Results.:**

Haitians showed the lowest coverage for demand for family planning satisfied with modern methods (44.2%), antenatal care (65.3%), skilled birth attendance (39.5%) and careseeking for suspected pneumonia (37.9%), and the highest for oral rehydration salts for diarrhea (52.9%), whereas Haitian migrants had the lowest coverage in DPT3 (44.1%) and oral rehydration salts for diarrhea (38%) and the highest in careseeking for suspected pneumonia (80.7%). Dominicans presented the highest coverage for most indicators, except oral rehydration salts for diarrhea and careseeking for suspected pneumonia. The composite coverage index was 79.2% for Dominicans, 69.0% for Haitian migrants, and 52.6% for Haitians. Socioeconomic inequalities generally had pro-rich and pro-urban pattern in all analyzed groups.

**Conclusion.:**

Haitian migrants presented higher coverage than Haitians, but lower than Dominicans. Both countries should plan actions and policies to increase coverage and address inequalities of maternal health interventions.

## RESUMO

**Objetivo.:**

Avaliar cobertura e desigualdades nas intervenções em saúde materno-infantil entre os haitianos, migrantes haitianos na República Dominicana e dominicanos.

**Métodos.:**

Estudo transversal utilizando dados de pesquisas representativas nacionalmente realizadas no Haiti em 2012, e na República Dominicana em 2014. Nove indicadores foram comparados: demanda por planejamento familiar atendida com métodos modernos, atendimento pré-natal, atendimento ao parto (presença de profissional qualificado no parto), vacinação de crianças (BCG, sarampo e DPT3), atendimento de crianças (sais de reidratação oral para diarreia e demanda por assistência por suspeita de pneumonia) e índice composto de cobertura. A riqueza foi medida por meio de índice baseado em recursos, dividido em tercis, e o local de residência (urbano ou rural) foi estabelecido segundo a definição dos países.

**Resultados.:**

Os haitianos apresentaram a menor cobertura de demanda por planejamento familiar atendida com métodos modernos (44,2%), atendimento pré-natal (65,3%), presença de profissional qualificado no parto (39,5%) e de atendimento por suspeita de pneumonia (37,9%), e a mais alta para sais de reidratação oral na diarreia (52,9%), enquanto os migrantes haitianos tiveram a menor cobertura de DPT3 (44,1%) e sais de reidratação oral para diarreia (38%), e a mais alta na assistência por suspeita de pneumonia (80,7%). Os dominicanos apresentaram a cobertura mais alta para a maioria dos indicadores, exceto para sais de reidratação oral para diarreia e demanda por assistência por suspeita de pneumonia. O índice composto de cobertura foi 79,2% para dominicanos, 69,0% para migrantes haitianos e 52,6% para os haitianos. De forma geral, as desigualdades socioeconômicas apresentaram padrão pró-riqueza e pró-urbano em todos os grupos analisados.

**Conclusões.:**

Os migrantes haitianos apresentaram maior cobertura que os haitianos, mas coberturas inferiores aos dominicanos. Ambos os países devem planejar ações e políticas para aumentar a cobertura e abordar as desigualdades nas intervenções em saúde materna.

La migración es un fenómeno universal que implica el desplazamiento de una zona geográfica a otra, y es considerada como uno de los cuatro mecanismos de la evolución biológica (1). En el 2015, alrededor de 250 millones de personas migraron en todo el mundo, y casi la mitad eran mujeres (2, 3). En América Latina y el Caribe, en ese mismo año emigraron 25 millones de personas a Estados Unidos y 4,6 millones a Europa. Además de esa migración interregional que está bien descrita, el flujo migratorio de Haití a la República Dominicana representa una de las rutas migratorias intrarregionales más prominentes del Caribe (2). Las Naciones Unidas informaron que 329 281 haitianos que emigraron a la República Dominicana en el 2015, cifra que fue un 57% mayor que la notificada en 1990 (4).

Las razones de esa migración son, entre otras, la inestabilidad política, la situación económica persistentemente precaria y los frecuentes desastres naturales en Haití (5). Según la primera encuesta de inmigrantes en la República Dominicana (ENI-2012), los migrantes constituían el 5,4% de la población total del país. El 87,3% de esos migrantes nacieron en Haití, lo que muestra el predominio de los migrantes haitianos en el país (6).

Las mujeres haitianas siempre han estado presentes en los flujos migratorios hacia el país vecino (6). Inicialmente, se desplazaban acompañando a la fuerza laboral masculina (7). Sin embargo, muchas de ellas tienen un acceso limitado a los servicios de salud sexual y reproductiva en la República Dominicana, porque es posible que no tengan la documentación necesaria o que se les nieguen sus derechos (8). Esta situación puede incidir negativamente en la salud física y emocional de las mujeres inmigrantes y sus hijos, colocándolos en una posición de mayor vulnerabilidad.

La meta 10.7 de los Objetivos de Desarrollo Sostenible subraya la necesidad de "facilitar la migración y la movilidad ordenadas, seguras, regulares y responsables de las personas, incluso mediante la aplicación de políticas migratorias planificadas y bien gestionadas" (https://www.un.org/sustainabledevelopment/es/objetivos-de-desarrollo-sostenible/). Pese al continuo desplazamiento de migrantes de Haití a la República Dominicana, poco se sabe sobre la manera en que su situación migratoria influye en la cobertura de las intervenciones en salud reproductiva, materna, neonatal e infantil. El objetivo de este estudio fue evaluar la cobertura y las desigualdades en las intervenciones de salud maternoinfantil, comparando las mujeres migrantes haitianas y sus hijos que viven en la República Dominicana con las haitianas y dominicanas que viven en sus respectivos países.

## MATERIALES Y MÉTODOS

Este es un estudio transversal, en el que se utilizaron datos de encuestas representativas a nivel nacional realizadas en Haití en el 2012 (Encuesta Nacional de Demografía y Salud, conocida como DHS por su sigla en inglés) y en la República Dominicana en el 2014 (encuesta de indicadores múltiples por conglomerados, http://mics.unicef.org/, conocida como MICS por su sigla en inglés). En ambas encuestas se recopiló información estandarizada sobre las características de los hogares, las personas y la comunidad, a fin de hacer una comparación entre los países (9). Se analizó la información recopilada de mujeres de 15 a 49 años y sus hijos menores de 5 años.

### Indicadores de cobertura

Se analizaron nueve indicadores de intervención en salud maternoinfantil. Estos indicadores siguieron las definiciones de la iniciativa Countdown to 2030 (http://countdown2030.org) y son: 1) demanda de planificación familiar satisfecha con métodos modernos (DPFSmm): proporción de mujeres de 15 a 49 años actualmente casadas o en unión libre que necesitan anticoncepción y que están utilizando (o cuya pareja está utilizando) un método anticonceptivo moderno; 2) cuatro o más consultas de atención prenatal (APN4+): proporción de mujeres de 15 a 49 años que dieron a luz en los tres años anteriores (según la encuesta DHS) o dos años anteriores (según la encuesta MICS) y que tuvieron por lo menos cuatro consultas de atención prenatal; 3) atención del parto por personal de salud calificado (APPC): proporción de mujeres de 15 a 49 años que dieron a luz en los tres años (según la encuesta DHS) o dos años anteriores (según la encuesta MICS) cuyo parto fue asistido por personal calificado; 4) vacunación infantil (BCG): proporción de niños vivos de entre 12 y 23 meses de edad que recibieron la vacuna BCG; 5) vacunación infantil (DPT3): proporción de niños de entre 12 y 23 meses de edad que recibieron tres dosis de la vacuna DPT (difteria, tos ferina y tétanos); 6) vacunación infantil contra el sarampión (VIS): proporción de niños de entre 12 y 23 meses de edad que recibieron la vacuna contra el sarampión (monovalente o no); 7) búsqueda de atención de salud por sospecha de neumonía (BASN): proporción de menores de 5 años vivos con sospecha de neumonía en las últimas dos semanas para los cuales se buscó tratamiento en un centro de salud o prestador adecuado; 8) administración de sales de rehidratación oral para la diarrea (SRO): proporción de menores de 5 años vivos con diarrea en las últimas dos semanas que recibieron tratamiento de rehidratación oral (paquetes de sales de rehidratación oral, recomendación de una solución casera o aumento de líquidos), y 9) índice de cobertura compuesto (ICC): promedio ponderado de los ocho indicadores mencionados, en relación con la continuidad de la atención. El índice de cobertura compuesto fue propuesto inicialmente por Boerma et al. en el 2008 (10) y fue actualizado luego por la iniciativa Countdown to 2030 (11) de la siguiente manera:

$$ICC=\frac{1}{4}\left(DPFSmm+\frac{APN4+APPC}{2}+\frac{BCG+2\times DPT3+VIS}{4}+\frac{SRO+BASN}{2}\right)$$

Todos los indicadores utilizados en estos análisis han sido estandarizados en el Centro Internacional para la Equidad en la Salud (ICEH, por su sigla en inglés; www.equidade.org), lo que permite hacer comparaciones entre las dos encuestas. Los procedimientos de estandarización aseguran que la definición de los indicadores, sus numeradores y denominadores sean congruentes en las diferentes encuestas.

### Situación migratoria

La situación migratoria se definió según el idioma primario hablado por la mujer o la persona jefe del hogar. Las mujeres y los niños fueron clasificados en tres grupos: 1) haitianos: los que fueron incluidos en la encuesta DHS de Haití; 2) dominicanos: los que fueron incluidos en la encuesta MICS de la República Dominicana e indicaron que su idioma principal era el español, y 3) migrantes haitianos: los que fueron incluidos en la encuesta MICS de la República Dominicana y su idioma principal era el creole (criollo haitiano), dado que este es el idioma principal que hablan los migrantes y uno de los idiomas oficiales de Haití.

### Estratificadores

En los análisis se incluyeron dos variables adicionales: el lugar de residencia y el índice de riqueza. El lugar de residencia se dividió en urbano y rural, según las definiciones de cada país. El índice de riqueza se basa en los activos de cada hogar. Se calcula mediante un análisis de los componentes principales y, en la puntuación, se consideran las diferencias entre los activos de los hogares urbanos y rurales. Los detalles del cálculo del índice de riqueza se pueden encontrar en otras publicaciones (12). Debido a las limitaciones del tamaño de muestra, la puntuación se dividió en terciles, donde el primer tercil (T1) representa el tercio más pobre de la muestra y el tercer tercil (T3) representa el tercio más rico. Los índices de riqueza se calcularon por separado para cada una de las dos encuestas.

### Análisis de datos

Las características socioeconómicas y demográficas de los dos países se obtuvieron de la base de datos del Banco Mundial (https://datos.bancomundial.org/) y del Index Mundi (https://www.indexmundi.com), ambos consultados en enero del 2020, con el fin de proporcionar una breve descripción de estos países.

Se estimó la cobertura de los nueve indicadores estudiados y su respectivo intervalo de confianza del 95% para cada uno de los tres grupos según su situación migratoria teniendo en cuenta el diseño de la muestra. Los análisis también se estratificaron por terciles según el índice de riqueza y el lugar de residencia, dentro de los grupos migratorios.

Se calculó para cada indicador el índice de desigualdad de la pendiente (IDP), una medición compleja de la desigualdad absoluta, en función de la regresión logística. El IDP representa la diferencia absoluta en puntos porcentuales con base en los valores predichos para un indicador determinado, entre los extremos superior e inferior del espectro socioeconómico. El IDP puede variar de -100 a 100 puntos porcentuales. Un valor de cero indica que no hay desigualdad; los valores positivos indican que la cobertura es mayor para las personas más ricas, en tanto que los valores negativos representan lo contrario. Todos los análisis se realizaron utilizando el programa estadístico Stata^®^, versión 15.1 (StataCorp LP, College Station, Texas, Estados Unidos).

### Aspectos éticos

Los organismos responsables obtuvieron la aprobación ética para cada encuesta. Los datos utilizados en los análisis por los equipos de MICS y DHS son anónimos y están disponibles públicamente.

## RESULTADOS

En el cuadro 1 se muestran las características socioeconómicas y demográficas de Haití y la República Dominicana. La superficie de la República Dominicana es casi el doble que la de Haití, pero con una menor densidad demográfica. La tasa de migración líquida, que se calcula restando el número de personas que emigran de las que inmigraron, es más baja en Haití (-6,9 por 1000 habitantes), que en la República Dominicana (-1,9 por 1000 habitantes), lo que indica que es mayor el número de haitianos que emigran que el número de inmigrantes que llegan al país. En general, los indicadores demográficos (esperanza de vida y mortalidad) y socioeconómicos (PIB per cápita y alfabetización) son mejores en la República Dominicana que en Haití.

**CUADRO 1.** Características demográficas y socioeconómicas de Haití y la República Dominicana

	Haití	República Dominicana
**Superficie total (km²)**	27 560	48 310
Rural	26 163 (94,9%)	42 784 (88,6%)
**Población total**	9 801 664	10 349 740
**Densidad de población** (habitantes/km²)	353,2	212,7
**Crecimiento demográfico** (crecimiento porcentual anual medio)	0,9	1,3
**Esperanza de vida**	62,5	77,8
**Tasa de natalidad** (por 1000)	23,9	19,0
**Tasa de mortalidad general** (por 1000)	8,1	4,5
**Tasa de migración líquida** (inmigración – emigración, por 1000)	-6,9	-1,9
**Tasa de mortalidad en menores de 1 año** (por 1000)	52,4	19,6
**Tasa de mortalidad materna** (por 100 000)	359	92
**Tasa de alfabetización**	60,7	91,8
**Producto interno bruto (PIB) per cápita** (US$, ajustado por la paridad del poder adquisitivo)	1 300	9 700
**PIB, tasa de crecimiento** (variación porcentual anual, ajustado por la inflación monetaria)	2,8	2,0

***Nota:*** Los datos se obtuvieron utilizando estimaciones del Banco Mundial (https://datos.bancomundial.org/) e IndexMundi (https://www.indexmundi.com/), consultados en enero del 2020, con las estimaciones más recientes disponibles desde el 2015.

**FIGURA 1.**
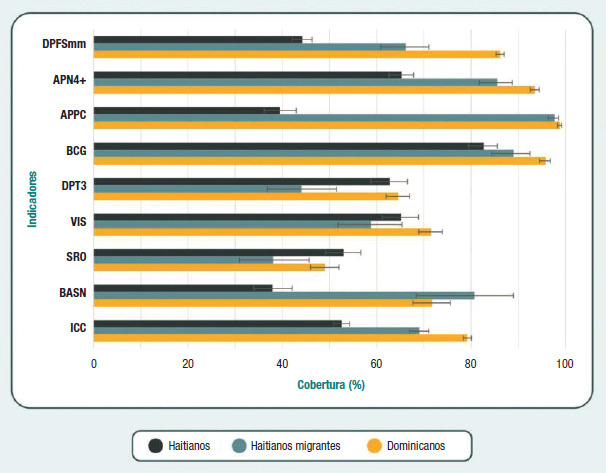
Cobertura de los indicadores de salud reproductiva, materna, neonatal e infantil en haitianos, haitianos migrantes y dominicanos

En la figura 1 se muestra la cobertura de las intervenciones de salud reproductiva, materna, neonatal e infantil para haitianos, haitianos migrantes y dominicanos. Las mujeres y los niños dominicanos presentaron la mayor cobertura de los tres grupos para la mayoría de los indicadores, que van desde 49,0% en la administración de sales de rehidratación oral para la diarrea hasta 98,6% en la atención del parto por personal de salud calificado, y no existe casi ninguna superposición entre el intervalo de confianza del 95%. Los dominicanos tuvieron una cobertura más baja en la búsqueda de atención de salud por sospecha de neumonía que los migrantes haitianos (71,8% y 80,7%, respectivamente). Los haitianos presentaron la cobertura más baja en relación con los otros grupos en los siguientes indicadores: demanda de planificación familiar satisfecha, cuatro o más consultas de atención prenatal, atención del parto por personal calificado, vacunación infantil con BCG y búsqueda de atención de salud por sospecha de neumonía, pero tuvieron la mayor cobertura en la administración de sales de rehidratación oral para la diarrea. La cobertura de vacunación (vacuna contra el sarampión y tres dosis de la vacuna DPT) y la administración de sales de rehidratación oral para la diarrea fueron más bajas en los migrantes haitianos. El índice resumido de las intervenciones de salud reproductiva, materna, neonatal e infantil, índice de cobertura compuesto, fue de 79,2% para los dominicanos; 69,0% para los migrantes haitianos y 52,6% para los haitianos.

DPFSmm, demanda de planificación familiar satisfecha con métodos modernos; APN4+, al menos cuatro o más consultas de atención prenatal; APPC, atención del parto por personal de salud calificado; BCG, vacunación infantil; VIS, vacunación infantil contra el sarampión; DPT3, tres dosis de la vacuna DPT; SRO, administración de sales de rehidratación oral para la diarrea; BASN, búsqueda de atención de salud por sospecha de neumonía; ICC, índice de cobertura compuesto.

**FIGURA 2.**
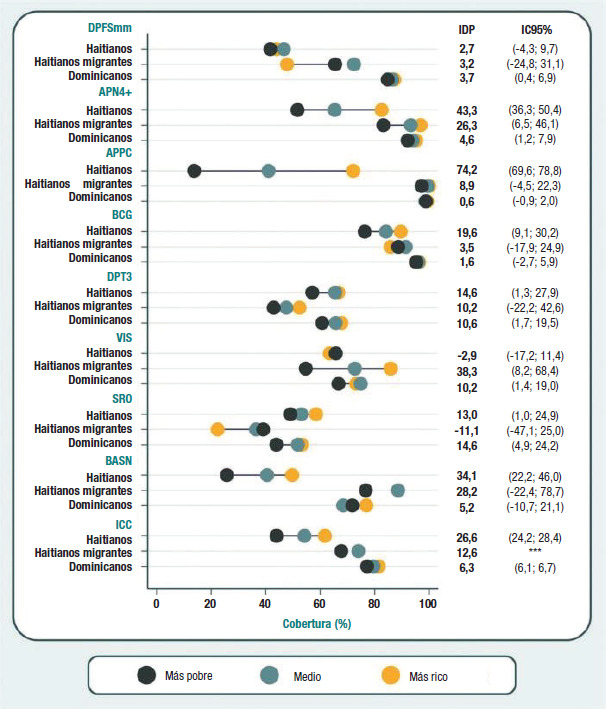
Cobertura de los indicadores de salud reproductiva, materna, neonatal e infantil por situación migratoria y tercil de riqueza

En la figura 2 se presenta la cobertura de los indicadores de atención de salud reproductiva, materna, neonatal e infantil por tercil de riqueza para cada grupo. En general, las mujeres y los niños pertenecientes al tercil más rico presentaron una mayor cobertura en comparación con los pertenecientes al tercil más pobre. Los valores positivos del IDP indican patrones de cobertura a favor de los ricos. En seis de los nueve indicadores, los haitianos presentaron el IDP más alto, en tanto que los dominicanos presentaron el IDP más bajo en cinco de los indicadores. La mayor desigualdad a favor de los ricos se observó en la atención del parto por personal de salud calificado en las mujeres haitianas (IDP = 74,2 puntos porcentuales). En el cuadro 2 se presenta el tamaño de las muestras y los intervalos de confianza del 95% (IC95%) para estas mediciones.

**FIGURA 3.**
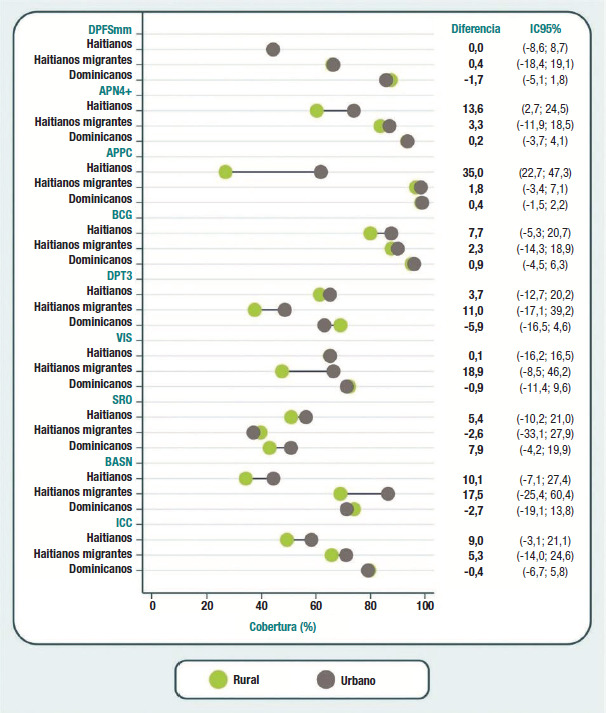
Cobertura de los indicadores de salud reproductiva, materna, neonatal e infantil por situación migratoria y lugar de residencia

En la figura 3 se muestran la cobertura de los indicadores de atención de la salud reproductiva, materna, neonatal e infantil de acuerdo con la situación migratoria y el lugar de residencia, y la diferencia absoluta entre zonas urbanas y rurales en puntos porcentuales. En general, las desigualdades tienen un patrón a favor de las zonas urbanas. Las mujeres haitianas presentaron las mayores desigualdades a favor de las zonas urbanas respecto de la cobertura de cuatro o más consultas de atención prenatal y atención del parto por personal de salud calificado (13,6 y 35,0 puntos porcentuales, respectivamente), en tanto que los migrantes haitianos mostraron las mayores desigualdades a favor de las zonas urbanas en cuanto a la vacunación infantil contra el sarampión y con la DPT3 (18,9 puntos porcentuales y 11,0 puntos porcentuales, respectivamente). Las desigualdades entre zonas urbanas y rurales del índice de cobertura compuesto fueron mayores para los haitianos y los migrantes haitianos (9,0 puntos porcentuales y 5,3 puntos porcentuales, respectivamente) que para los dominicanos (-0,4 puntos porcentuales). En el cuadro 2 se presenta el tamaño de las muestras y los IC95% por residencia urbana o rural.

## DISCUSIÓN

Los resultados indican que, en general, la cobertura de las mujeres y niños haitianos migrantes en la República Dominicana es mayor que para los haitianos que residen en su país de origen. Sin embargo, a pesar de residir en el mismo país, los migrantes haitianos siguen teniendo menor cobertura que los dominicanos. Se encontraron desigualdades en los haitianos y los migrantes haitianos, pero los primeros presentaron disparidades más amplias para la mayoría de los indicadores.

DPFSmm, demanda de planificación familiar satisfecha con métodos modernos; APN4+, al menos cuatro o más consultas de atención prenatal; APPC, atención del parto por personal de salud calificado; BCG, vacunación infantil; VIS, vacunación infantil contra el sarampión; DPT3, tres dosis de la vacuna DPT; SRO, administración de sales de rehidratación oral para la diarrea; BASN, búsqueda de atención de salud por sospecha de neumonía; ICC, índice de cobertura compuesto

En el 2001, el gobierno de la República Dominicana aprobó una reforma estructural del sistema de salud (Ley 87-01), que incluyó un plan de beneficios para toda la población con el fin de lograr una cobertura universal y equitativa que reflejara un modelo de salud inclusivo. La reforma de la salud establecía que las personas pobres serían subsidiadas mediante pagos per cápita financiados con los impuestos generales (13). Esto puede explicar parcialmente la mayor cobertura de atención de salud reproductiva, materna, neonatal e infantil observada en la República Dominicana, tanto para mujeres y niños nativos como inmigrantes.

En contraste, solo la mitad de la población haitiana tiene acceso regular a la atención de salud, principalmente debido a la pobreza generalizada, la escasez de profesionales de la salud en el país (14), la escasez de inversión crónica en infraestructura de salud y la inestabilidad política (5).

Agravando la crisis preexistente, el fuerte terremoto del 2010 perjudicó el acceso a los servicios básicos en Haití, como la atención de la salud, la educación y el acceso a agua potable, así como el empleo y otras oportunidades de generación de ingresos (15). Después de un desastre natural pueden producirse interrupciones en los servicios de salud, lo que afecta las intervenciones rutinarias de salud reproductiva, materna, neonatal e infantil, dado que las prioridades médicas se reorientan hacia la atención de emergencia (5). Después del terremoto aumentó considerablemente la ayuda humanitaria extranjera; se recibieron cuantiosas donaciones de recursos financieros e insumos médicos por parte de instituciones internacionales. Tohme et al. constataron que Haití mejoró significativamente sus servicios de vacunación y la vigilancia de enfermedades prevenibles mediante vacunación durante el período comprendido entre el 2010 y el 2016 (16). Esto puede explicar la mayor cobertura de vacunación (vacuna contra el sarampión y tres dosis de la vacuna DPT) en los niños haitianos en comparación con los migrantes haitianos. La vacunación es una intervención relativamente sencilla de ofrecer a nivel comunitario, sin necesidad de las complejas infraestructuras que requieren otras intervenciones, como la atención del parto en un establecimiento de salud.

DPFSmm, demanda de planificación familiar satisfecha con métodos modernos; APN4+, al menos cuatro o más consultas de atención prenatal; APPC, atención del parto por personal de salud calificado; BCG, vacunación infantil; VIS, vacunación infantil contra el sarampión; DPT3, tres dosis de la vacuna DPT; SRO, administración de sales de rehidratación oral para la diarrea; BASN, búsqueda de atención de salud por sospecha de neumonía; ICC, índice de cobertura compuesto.

En general, la bibliografía ha mostrado que las mujeres migrantes tienen peor cobertura de salud que las residentes del país receptor (17-19). Existen varias razones posibles, como los obstáculos lingüísticos (especialmente al llegar), la discriminación, la falta de documentos, los costos de transporte y la distancia entre el lugar de residencia y los servicios de salud, entre otras (20-24). Por ejemplo, poseer documentos personales es esencial para todos y aún más para los inmigrantes. Al no contar con documentos, los inmigrantes pueden enfrentar restricciones en el acceso a la atención médica, la educación y la movilidad laboral en el país (6, 8, 22). Estos factores pueden llevar a las personas a buscar servicios de urgencia en lugar de servicios de salud preventiva basados en la atención primaria, como se ha observado en otros contextos migratorios (25).

**CUADRO 2.** Tamaños de muestra e intervalo de confianza del 95% para cada uno de los indicadores, por situación migratoria, tercil de riqueza y lugar de residencia

Indicador	Grupo		Tercil de riqueza			Lugar de residencia	
			Más pobre	Medio	Más rico	Urbano	Rural
**Mujeres**							
Demanda de planificación familiar (con métodos modernos)	Haitianos	N	1974	2075	1521	2248	3323
		Estimación del IC95%	38,3-35,2	43,2-50,2	40,8-47,2	41,3-47,3	41,3-47,2
	Haitianos inmigrantes	N	771	102	19	402	491
		Estimación del IC95%	60,5-70,2	57,9-83,4	16,7-80,7	58,3-73,5	60,0-71,4
	Dominicanos	N	5290	4304	3607	8463	4738
		Estimación del IC95%	83,2-86,2	84,8-87,8	85,6-88,7	84,7-86,7	85,9-88,8
Cuatro o más consultas de atención prenatal	Haitianos	N	1701	1480	862	1393	2650
		Estimación del IC95%	47,7-55,7	61,9-68,6	78,8-85,7	70,2-77,2	56,7-63,7
	Haitianos inmigrantes	N	598	70	15	322	361
		Estimación del IC95%	78,6-86,9	84,0-97,3	77,4-99,6	82,3-90,5	76,6-88,9
	Dominicanos	N	3033	2154	1628	4538	2277
		Estimación del IC95%	90,3-93,6	92,3-95,2	93,3-96,4	92,4-94,6	91,7-94,8
Atención del parto por personal de salud calificado	Haitianos	N	1937	1669	934	1539	3001
		Estimación del IC95%	11,5-16,4	37,4-44,8	67,8-76,1	57,1-66,2	23,2-30,6
	Haitianos inmigrantes	N	598	70	15	322	361
		Estimación del IC95%	95,4-98,3	95,8-99,9	^*^	96,7-99,3	93,8-98,2
	Dominicanos	N	3033	2154	1628	4538	2277
		Estimación del IC95%	97,9-99,3	97,4-99,2	98,2-99,7	98,2-99,4	97,7-99,1
**Niños**							
Vacuna BCG	Haitianos	N	567	518	285	471	899
		Estimación del IC95%	70,2-81,6	79,4-88,0	85,0-92,8	83,5-90,8	75,3-83,9
	Haitianos inmigrantes	N	296	43	8	171	176
		Estimación del IC95%	83,3-92,2	74,1-97,5	41,5-98,1	82,8-94,3	80,6-92,4
	Dominicanos	N	1454	1077	837	2246	1132
		Estimación del IC95%	92,4-97,0	94,1-97,3	94,0-97,7	94,7-97,0	91,3-97,3
Vacuna contra el sarampión	Haitianos	N	567	518	285	471	899
		Estimación del IC95%	58,1-72,6	60,5-70,6	56,7-69,9	59,4-70,6	59,8-70,0
	Haitianos inmigrantes	N	289	43	8	169	171
		Estimación del IC95%	46,7-62,5	55,1-85,3	41,5-98,1	57,7-74,0	57,7-74,0
	Dominicanos	N	1437	1066	823	2209	1117
		Estimación del IC95%	62,8-70,4	70,6-78,7	68,1-77,8	68,2-74,1	67,4-76,4
Vacuna DPT (tres dosis)	Haitianos	N	567	518	285	471	899
		Estimación del IC95%	50,3-63,6	59,7-71,0	60,1-72,6	59,1-70,7	56,2-66,4
	Haitianos inmigrantes	N	285	43	8	167	169
		Estimación del IC95%	35,1-51,2	30,6-65,2	18,9-83,9	38,4-58,8	28,3-47,6
	Dominicanos	N	1422	1059	825	2201	1105
		Estimación del IC95%	56,8-64,5	61,2-69,8	62,9-72,3	60,0-66,0	64,3-73,3
Administración de sales de rehidratación oral	Haitianos	N	596	565	254	488	927
		Estimación del IC95%	43,7-54,5	47,1-59,0	51,4-65,3	50,2-62,2	46,2-55,6
	Haitianos inmigrantes	N	276	37	6	156	163
		Estimación del IC95%	31,2-47,6	20,0-56,8	4,9-61,8	28,2-46,8	28,3-52,2
	Dominicanos	N	1500	969	609	2063	1015
		Estimación del IC95%	40,4-47,6	45,9-57,7	4,.7-59,0	47,2-54,4	38,5-47,5
Búsqueda de atención de salud por sospecha de neumonía	Haitianos	N	433	373	202	332	676
		Estimación del IC95%	20,4-32,0	34,4-46,9	42,9-56,5	38,7-50,2	29,1-39,8
	Haitianos inmigrantes	N	53	14	^**^	34	33
		Estimación del IC95%	61,1-87,4	64,0-97,1	^**^	71,2-94,2	48,4-84,0
	Dominicanos	N	491	365	192	715	333
		Estimación del IC95%	65,8-77,1	62,0-74,2	65,5-85,5	66,3-75,7	66,8-79,9
**Indicador combinado**							
Índice de cobertura compuesto	Haitianos		^***^	^***^	^***^	^***^	^***^
		Estimación del IC95%	40,9-47,0	52,0-56,4	59,9-63,6	56,2-60,3	46,9-51,8
	Haitianos inmigrantes	N	^***^	^***^	^***^	^***^	^***^
		Estimación del IC95%	65,2-70,3	69,2-78,8	^**^	68,7-73,3	62,3-69,2
	Dominicanos	N	^***^	^***^	^***^	^***^	^***^
		Estimación del IC95%	76,0-78,4	78,5-80,7	80,2-82,7	78,2-80,0	78,1-80,9

BGC, *Bacillus* Calmette Guérin; DPT, difteria, tétanos y tos ferina; IC95%, intervalo de confianza del 95%.

* La cobertura es del 100%.

** Datos no disponibles.

*** Dado que el índice de cobertura compuesto es una medida ponderada calculada al nivel del grupo, no se calcula el valor de N.

La aculturación es un importante proceso social que explica los determinantes y las consecuencias de las disparidades en materia de salud en las poblaciones minoritarias (26) porque es la manera en la cual los inmigrantes interiorizan la cultura mediante la adopción de las normas, los valores y las prácticas de su nuevo lugar de residencia (23). Por lo tanto, el hecho de que la cobertura de la salud reproductiva, materna, neonatal e infantil en los migrantes haitianos sea menor que en los dominicanos, pero mayor que en los haitianos, podría deberse en parte a cierto nivel de aculturación de los migrantes y a una mejor calidad de la atención médica que la que disponen en Haití. Desafortunadamente, no fue posible explorar con más detalle el nivel de aculturación en nuestro estudio debido a la falta de información sobre el tiempo transcurrido entre la fecha en que las personas inmigraron a la República Dominicana y la fecha en que se realizó la encuesta.

Dos indicadores de cobertura merecen una atención especial: la administración de sales de rehidratación oral para la diarrea y la búsqueda de atención de salud por sospecha de neumonía. El acceso al agua potable puede verse amenazado por desastres naturales, con un mayor riesgo de brotes de enfermedades diarreicas (27). Después del terremoto del 2010 hubo un brote de cólera, que afectó particularmente a las zonas rurales de Haití. La respuesta a la epidemia incluyó la distribución comunitaria de sales de rehidratación oral y sistemas de purificación de agua, y la educación sobre prácticas de higiene, entre otras intervenciones. Uno de los cinco mensajes básicos que difundieron los medios de comunicación en ese momento fue administrar sales de rehidratación oral a cualquier persona con diarrea (14, 28, 29), además de la provisión generalizada de paquetes de sales para ese fin por parte del gobierno y organizaciones voluntarias. Esto puede explicar por qué el uso de sales de rehidratación oral fue mayor en Haití que en la República Dominicana, donde la epidemia fue menos grave en los dominicanos y los inmigrantes (14).

La búsqueda de atención de salud por sospecha de neumonía fue el único indicador para el que los migrantes haitianos presentaron la mayor cobertura. Sin embargo, este resultado debe interpretarse con cuidado ya que este indicador específico se analizó con el tamaño de muestra más pequeño (N=67), y fue el único con menos de 100 mujeres o niños en el denominador. El intervalo de confianza del 95% para esta estimación varió entre el 68,4% y el 88,9%.

Tanto el lugar de residencia como la riqueza pueden desempeñar un papel importante en la cobertura de las intervenciones de salud maternoinfantil. Las desigualdades en la cobertura con intervenciones en salud reproductiva, materna, neonatal e infantil suelen mostrar patrones a favor de los ricos y de las zonas urbanas (30). La evidencia indica que las desigualdades socioeconómicas preexistentes suelen exacerbarse en casos de desastres (5), lo que puede explicar por qué las disparidades fueron tan amplias en Haití. La residencia en zonas rurales dificulta el acceso a establecimientos de salud adecuados debido a los obstáculos geográficos y económicos (31, 32). En este estudio, las mujeres y los niños de zonas urbanas tuvieron mayor cobertura que los de zonas rurales en los tres grupos estudiados. La brecha entre zonas urbanas y rurales fue más estrecha entre los dominicanos, probablemente debido a la existencia de un sistema de salud más estructurado y descentralizado en la República Dominicana (13), con menos obstáculos al acceso para los dominicanos y para los migrantes haitianos, a diferencia de la situación de los haitianos que viven en Haití (33).

Los análisis desglosados por situación migratoria y por riqueza facilitaron la evaluación de la interseccionalidad. Estos resultados sugieren que las desigualdades relacionadas con la riqueza son mucho más amplias en Haití que entre los dominicanos o los migrantes que residen en la República Dominicana. La existencia de las redes de protección social que se acaban de describir en la República Dominicana, aunadas a mejoras en otros determinantes sociales, pueden explicar por qué las diferencias socioeconómicas son menos marcadas que en Haití.

Este estudio tiene algunas limitaciones más allá de las ya mencionadas. En primer lugar, se utilizó el idioma que habla la mujer o la persona jefe de la casa como variable sustitutiva de la situación migratoria. Por lo tanto, no fue posible diferenciar a los inmigrantes aculturados, que posiblemente hayan indicado que el español era su lengua primaria, en lugar del creole (criollo haitiano), de los inmigrantes recién aculturados, teniendo en cuenta que no había información disponible sobre el tiempo que habían pasado en el país receptor. Sin embargo, el uso del idioma como variable sustitutiva de la situación migratoria es una alternativa fiable en las encuestas poblacionales (6). El pequeño tamaño de la muestra en algunos grupos también es una limitación. Aunque el tamaño de la muestra de mujeres y niños migrantes haitianos era de alrededor de 1000 individuos, los análisis que utilizaban una doble estratificación por riqueza y situación migratoria dieron lugar a tamaños pequeños de muestra, sobre todo para indicadores relacionados con el manejo de enfermedades que sólo se calculan para niños con una enfermedad reciente (30).

Este estudio también presenta algunas fortalezas. Son pocos los estudios sobre los efectos de la migración sobre la salud en países de ingresos bajos y medianos, ya que son pocas las encuestas que recopilan esa información (34). Las dos encuestas comparables realizadas durante un período de dos años permitieron realizar una comparación de las mujeres y los niños en sus países de origen y de destino. Además, la gama de indicadores incluidos en los análisis proporciona una visión general amplia y sólida de la cobertura de salud reproductiva, materna, neonatal e infantil (35). Otra fortaleza es que la presentación de resultados responde al Objetivo de Desarrollo Sostenible 17.18 que requiere el desglose de los indicadores de salud y otros indicadores conexos en varias dimensiones de la desigualdad, como la situación migratoria, la riqueza y el lugar de residencia (https://www.un.org/sustainabledevelopment/es/objetivos-de-desarrollo-sostenible/).

## CONCLUSIÓN

La situación migratoria de las mujeres y los niños afecta la salud de las madres y sus hijos, aunque los migrantes haitianos que residen en la República Dominicana presentan mejores indicadores que los haitianos que permanecieron en su país de origen. Es de esperar que estos resultados contribuyan a crear conciencia en los responsables de la formulación de políticas del Caribe, en particular en los dos países vecinos, sobre la importancia de documentar la salud de los migrantes y de realizar análisis estratificados para orientar la reducción de las desigualdades de salud, sin que nadie se quede atrás.

## Declaración.

Los autores son los únicos responsables de las opiniones expresadas en el manuscrito, que no necesariamente reflejan la opinión o las políticas de la *RPSP/PAJPH*, de la OPS o de la Universidad Federal de Pelotas.
